# Rodent Animal Models of Endometriosis-Associated Pain: Unmet Needs and Resources Available for Improving Translational Research in Endometriosis

**DOI:** 10.3390/ijms24032422

**Published:** 2023-01-26

**Authors:** Miguel A. Tejada, Carles Antunez, Paulina Nunez-Badinez, Bianca De Leo, Philippa T. Saunders, Katy Vincent, Antonio Cano, Jens Nagel, Raul Gomez

**Affiliations:** 1 Research Unit on Women’s Health-INCLIVA, Institute of Health Research, 46010 Valencia, Spain; 2 Bayer AG. Research & Early Development, Pharmaceuticals, Exploratory Pathobiology, Aprather Weg 18a, 42096 Wuppertal, Germany; 3 Bayer AG. Research & Early Development, Pharmaceuticals, Reproductive Health, Müllerstr. 178, 13342 Berlin, Germany; 4 Centre for Inflammation Research, Queen’s Medical Research Institute, The University of Edinburgh, 47 Little France Crescent, Edinburgh EH16 4TJ, UK; 5 Nuffield Department of Women’s and Reproductive Health, University of Oxford, Oxford OX3 9DU, UK; 6 Department of Pediatrics, Obstetrics and Gynecology, University of Valencia, 46010 Valencia, Spain; 7 Department of Pathology, University of Valencia, 46010 Valencia, Spain

**Keywords:** endometriosis models, pain, reflex and non-reflex test

## Abstract

Chronic pain induced by endometriosis is a maladaptive pain experienced by half of women with this disease. The lack of pharmacological treatments suitable for the long-term relief of endometriosis-associated pain, without an impact on fertility, remains an urgent unmet need. Progress has been slowed by the absence of a reproducible rodent endometriosis model that fully replicates human physiopathological characteristics, including pain symptoms. Although pain assessment in rodents is a complicated task requiring qualified researchers, the choice of the behavioral test is no less important, since selecting inappropriate tests can cause erroneous data. Pain is usually measured with reflex tests in which hypersensitivity is evaluated by applying a noxious stimulus, yet this ignores the associated emotional component that could be evaluated via non-reflex tests. We conducted a systematic review of endometriosis models used in rodents and the number of them that studied pain. The type of behavioral test used was also analyzed and classified according to reflex and non-reflex tests. Finally, we determined the most used reflex tests for the study of endometriosis-induced pain and the main non-reflex behavioral tests utilized in visceral pain that can be extrapolated to the study of endometriosis and complement traditional reflex tests.

## 1. Introduction

Endometriosis is a chronic inflammatory disorder produced by the implantation and growth of functional endometrial tissue at extrauterine sites. The disease is estimated to affect 10% of women of reproductive age [1,2,3,4], which represents about 193 million women worldwide [5]. Around 50% of those with endometriosis show associated symptoms, including pelvic pain [6,7] and/or infertility [8]. Chronic pain is a disabling symptom of disease, present in approximately 50–60% of affected women [2] and has a high impact on quality of life [9,10], as well as elevated socioeconomic costs in health care expenditure [11,12,13].

Endometriosis is a progressive disease, but diagnosis is often delayed by 7–10 years after symptoms onset [14] worsening the patient condition [15]. Although great efforts are being made for early and non-invasive diagnosis, no biomarkers capable of robustly detecting endometriosis are currently known [16,17,18].

Despite advances in research into the mechanism of action, therapeutic tools against endometriosis are still far from optimal. There is currently no cure for this pathology, and pharmacological treatments are mostly intended to alleviate symptoms and interfere with disease progression. Pain is the most distressing symptom associated with this pathology, the principal reason for doctor’s appointment, and thus the main clinical end point of interest. The clinical treatment of endometriosis-associated pain is variable, not always effective and limited by side-effects. Treatments of choice will depend on the severity of symptoms and the extent and location of the lesion. Most commonly, drugs that interfere with hormonal support are the first choice prescribed to alleviate pain as estrogens are required for lesion growth and activity. Side effects of antiestrogenic therapy are well known: induction of a pseudomenopausal state that cannot be prolonged over time [16,18].

Inflammation is assumed to contribute to the pain sensation through the irritating actions of proinflammatory cytokines, such as prostaglandins released in the peritoneal fluid by infiltrating immune cells [19,20], as well as by the ectopic endometrial tissue itself. Cytokines, such as TNF-α, IFN-γ, L-6, IL-8, IL-1β, IL-10, and VEGF, among others [21,22,23,24], also contribute to the perpetuation of the pathological situation by recruiting further immune cells and by promoting the adhesion, implantation, and survival of ectopic endometrial cells [23]. When such an inflammatory situation persists over time, the manifestation of continuous pain can cause the sensitization of nociceptive neurons, thus leading to the development of chronic pain [25]. In line with this, treatments to reduce endometriosis-associated pain are intended to abolish the noxious effect caused by proinflammatory cytokines. The most widely non-hormonal drugs used for such purposes are NSAIDs (see Table 1) [23,25]. However, when pain is not mediated by peripheral mediators of inflammation, but rather by sensitization at the central nervous system level, neuromodulator drugs (such as gabapentin or opioids) are used for endometriosis treatment [16]. The latter, however, are not free of serious side effects and only to minimize symptoms [18,26].

Surgery is especially indicated in cases refractory to pharmacological intervention [18,23,25,27] (see Table 1) and notably effective in interrupting pain sensation [28,29]. Unfortunately 15–20% of patients can develop postsurgical pain [30,31], and the recurrence rate of lesions is about 40–50% after 5 years [32,33].

**Table 1:** Summary of endometriosis treatment.

Treatment		Mechanism of Action	Side Effects	Ref.
**Hormonal treatment**	Combined oral contraceptives and progestins	Ovulation and decidualization suppression	Erratic bleeding, nausea, headaches, breast tenderness	[16,18,23,34]
	Progesterone	Produce endometrial atrophy inducing decidualization of endometrial cells	Abnormal uterine bleeding, vomiting, breast discomfort, and depression	[18,35,36]
	Gonadotrophin releasing Hormone (GnRH) agonists	Ovarian suppression	Vaginal dryness, hot flushes, reduced bone mineral density, mood instability	[16,18,23,34]
	Gonadotrophin releasing Hormone (GnRH) antagonists	Ovarian suppression	Vaginal dryness, hot flushes, reduced bone mineral density	[16,18,34]
	Aromatase inhibitors	Blocking estrogen produced locally in endometriotic lesions	Vasomotor symptoms, decreased libido, loss of calcium in the bones, nausea, and headaches	[16,18,23,34]
**Non-hormonal treatment**	NSAIDs	COX2 inhibitors	Potential ulcers, bleeding, perforation of the stomach and intestine, increased risk of heart attack, stroke, and related cardiovascular conditions	[16,18,23,34,37]
	Neuromodulatory drugs	Central nervous system desensitization	Dizziness, tiredness, drowsiness, change in mood, visual disturbance, shortness of breath, among others	[16,18,34,38]
**Surgery**		Remove the lesion by surgery	Possibility of disease recurrence and development of postsurgical pain	[18,23,32,33]

In this context, there remains an urgent unmet medical need for novel pharmacological targets to develop new, more effective drugs with fewer side effects to combat endometriosis. A requirement to properly address this task is the implementation of reliable and reproducible pre-clinical models able to reproduce the physiopathological characteristics of the disease, especially pain. 

Although endometriosis only appears spontaneously in menstruating primates, there is a need to model clinical aspects of the disease in rodents for pragmatical and ethical purposes. Mimicking endometriosis in mice brings advantages associated to the ease of use, low cost, deep understanding of rodent biology, possibility of using transgenic animals, and ability to perform tests with a higher number of individuals [39,40,41,42]. Some drawbacks can be found, however, such as physiological differences (absence of menstrual cycle, among others) and the phylogenetic distance between rodents and humans. As a consequence, species-specific effects might appear in such a way that the effectiveness of some drugs tested in rodents may not translate to humans. Nonetheless, endometriosis model development in rodents is expected to be beneficial for advancing disease understanding and developing potential effective treatments. In line with this, initiatives, such as the IMI-PainCare or WERF harmonization projects, have been launched to improve the face and generated validity of rodent endometriosis models.

Research into pain using experimental animals is commonly performed through a battery of behavioral tests evaluating responses to noxious stimuli [43]. However, pain is a more complex sensation than a mere physical response to a painful stimulus. Indeed, pain can be generated in the absence of external stimuli, and there is also an emotional sensation related to the affectation of well-being [44]. Moreover, those who suffer from endometriosis may present with pain-associated symptoms, including fatigue, anxiety, and depression [16]. During the last years, a series of behavioral non-reflex tests have emerged, in an attempt to identify this sick feeling and non-evoked manifestations of pain in rodents. Overall, such tests are aimed to quantify discomfort and fatigue-like symptoms and/or behaviors related to the reduction of social interactions as one would expect to happen in humans suffering pain [43].

For all of these reasons, rodent models of endometriosis used for translational research purposes would need to complement the evaluation of the physical component of pain by studying other aspects of behavioral responses (emotional factors), thus obtaining results that are more comparable to clinical ones. This literature review first summarizes the most widely used endometriosis models in rodents with their benefits and limitations, and second, focuses on how many of them measured pain and what type of behavioral tests were used. Our aim is to provide an overview of endometriosis rodent models and the main reflex tests used to evaluate the disease-related pain. Due to the infrequent use of non-reflexive tests in endometriosis models, we describe the most used non-reflexive tests on visceral pain applicable for the study of endometriosis.

## 2. Results

### 2.1. Endometriosis Models in Rodents: Limits and Solutions

**Figure 1:**
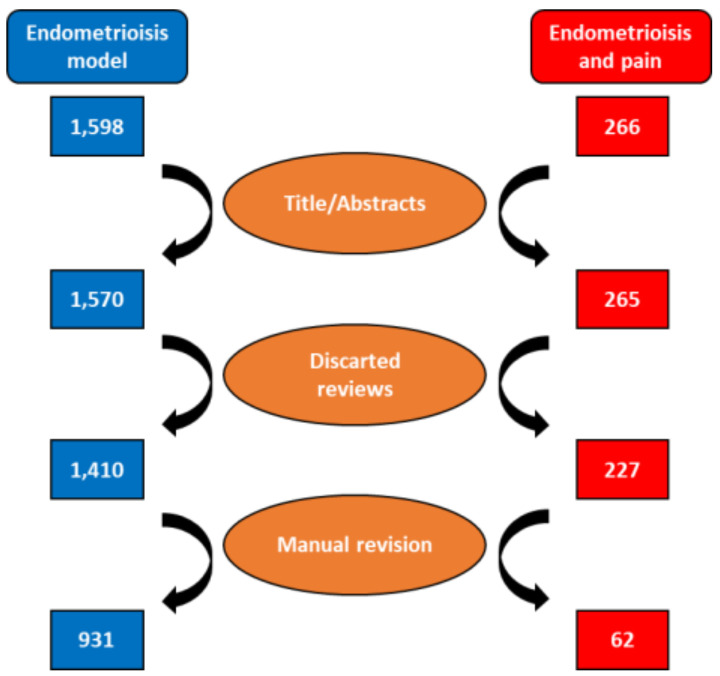
Flowchart of manuscript selection. The left column represents the total number of articles that studied endometriosis in rodents, while the right column shows the total number of articles that studied pain in rodent models of endometriosis.

In total, we retrieved 1598 papers published over the last 25 years with the parameters described in materials and methods. After limiting the results to Title/Abstract (1570) and removing the reviews (160), the number of works was reduced to 1410. Following manual review, the final number of papers was 931 (Figure 1).

**Figure 2:**
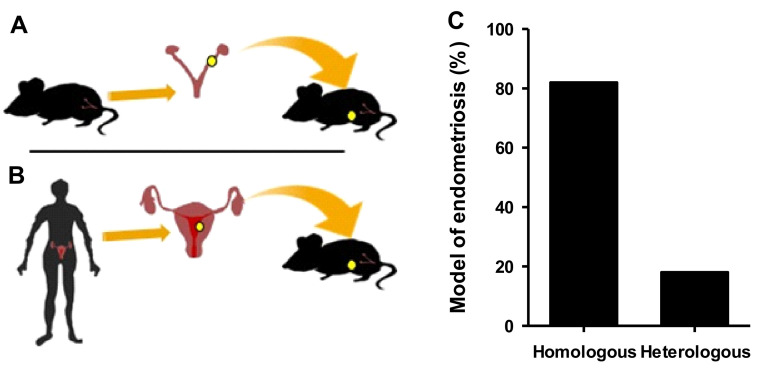
Endometriosis models in rodents. Schematic representation of homologous (**A**) and heterologous (**B**) endometriosis models. (**C**) Percentage of articles reporting homologous vs. heterologous disease induction in these two main models of endometriosis from 931 papers reviewed.

Two model types were differentiated depending on whether disease was homologously or heterologously induced (Figure 2A,B). After manually reviewing the articles in which endometriosis was induced in rodents, the kind of model used was analyzed. Most manuscripts used the homologous (82%) versus the heterologous (18%) model (Figure 2C). The characteristics and differences between these two models are detailed below. 

#### 2.1.1. Homologous Models

Homologous models (allograft) of endometriosis are obtained by implanting a small portion of uterus tissue at the intestinal mesenteric vessels and/or at the peritoneal wall or injecting resuspended tissue into the peritoneal cavity from the donor to recipient animal. The most commonly used method is inserting a fragment of the uterine horn from the donor (autologous or from in-bred strain to avoid host graft reaction), which is sutured or glued with tissue adhesive to the recipient [45]. 

Among the advantages of homologous models for endometriosis are their immunocompetence, expected suitability for long-term studies (i.e., no implant rejection) and non-dependence on obtention of human eutopic/ectopic endometrial tissue as a donor tissue. Moreover, it seems plausible to expect a similar pattern of altered cytokine and chemokine expression to that of human endometriosis [46]. In contrast, the main criticism of homologous models in terms of construct-validity is the limited similarity with humans related to endometrial physiology, as rodents do not menstruate, but rather have short estrous cycles without spontaneous decidualization. To overcome this limitation, several groups have manipulated the hormonal cycle of the mouse and used stimuli to induce decidualization in the uterus of donor mice, which is recovered and transferred into recipient mice either as decidualized or menstrual tissue. The lesions formed by these tissue samples were reported to phenocopy features of human endometriosis lesions [47,48,49,50]. 

Criticisms of homologous mouse models of endometriosis are centered on the fact that implanted ectopic uterine mouse tissue (or its decidualization) does not fully reflect the characteristics of human endometriotic lesions [51]. Nevertheless, given that this model mimics the pathophysiological hallmarks of the disease, such as gland and stroma epithelium in the lesions [49,50,52], as occurs in humans, homologous models are a good tool to improve the understanding of the disease.

#### 2.1.2. Heterologous Models

The heterologous model (xenograft) is obtained by implanting a portion of an endometrial biopsy from a human donor into an immunosuppressed mouse [52,53]. Although the implantation rate is around 30%, this percentage can increase to 100% when implants are sewn or glued using tissue adhesive [52]. Traditionally, the most widely used mice in these types of studies have been the athymic (Nude-NU), which lack mature T cells. However, although the mice are immunosuppressed, implants are not functional beyond the fourth week, due to lymphocytes observed in the third week [52]. One solution employed to avoid these problems is to use other rodent strains, such as SCID (severe combined immunodeficient) or NOD/SCID (non-obese diabetic severe combined immunodeficient) mice, which lack both T cells and functional B cells. In these severely immunosuppressed mice, the implantation rate is higher than that in athymic mice and implants are functional for more than 4 weeks [52]. Due to the role of other immune cells, such as NK and macrophages, NOD/SCID/γCnull (NOG) or RAG2/CD47/IL2RG mice were developed lacking B, T, and natural killer cell lymphocytes, and with impaired dendritic and macrophage cell functions [53]. 

The main advantages of the heterologous model over the homologous model in terms of construct validity is the possibility of using human eutopic or ectopic endometrium as a donor tissue for the development of lesions. However, a key limitation of these models is the use of immunosuppressed mice as recipients, so that a full evaluation of immune responses that play a role in the etiology of the disorder cannot be assessed. To try to reduce this deficiency, attempts have been made to administer immune cells from donors themselves [39].

### 2.2. Pain Assessment

Pain cannot be evaluated directly in rodents as they cannot verbally express the sensation of pain, creating a need to develop tests that quantify pain-associated behavior in animals [54]. Conventional pain assessment devices are focused on measuring animal responses upon exposure to a noxious stimulus, where the main responses evaluated are withdrawal, abdominal stretching, or licking [43]. However, in recent years, a growing number of studies has examined the emotional component of pain through non-evoked tests. In these kinds of tests, pain is assessed based on the decrease or absence of natural behavior in rodents. In this review, we describe the most used reflex and non-reflex tests in preclinical models for endometriosis and visceral pain. In addition, at the end of the different tests described, a summary table is included detailing how effective these tests are in the reviewed articles (Table 2).

#### 2.2.1. Study of Endometriosis-Induced Pain in Rodents

**Figure 3:**
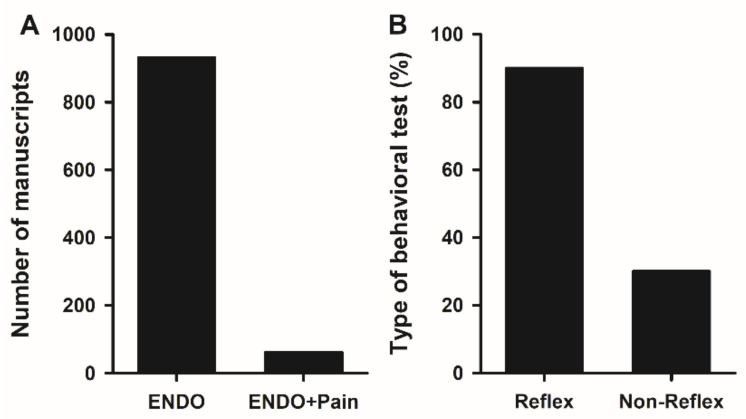
Preclinical model of endometriosis and pain. (**A**) Number of research articles that evaluated pain compared to all articles studying endometriosis in rodents. (**B**) Percentage of research articles that evaluated endometriosis-associated pain using reflex vs. non-reflex tests.

Applying the search parameters in the Medline database yielded 265 manuscripts with results included in the Title/Abstract. After discarding the reviews (n = 38) and performing a manual review, only 62 papers were found to have studied endometriosis-associated pain (Figure 1). These data are interesting because the main symptom in those who suffer from endometriosis is pain, and their greatest wish is pain eradication, or at least, reduction to manageable levels. Therefore, it is surprising to see that only 6.6% (Figure 3A) of studies addressing this pathology in rodents focused on pain. Most research groups aimed to reduce or eradicate endometrial lesions via different mechanisms but omitted any observations regarding pain development.

To further analyze the type of behavioral test used to measure endometriosis-induced pain, tests were stratified into two groups depending on whether the applied stimulus evokes a reflex or non-reflex response. About 90% of articles used reflex tests to study this kind of pain, and more than 70% employed this type of test alone. Only 30% of publications used non-reflex tests to complement selected reflex tests (Figure 3B).

**Figure 4:**
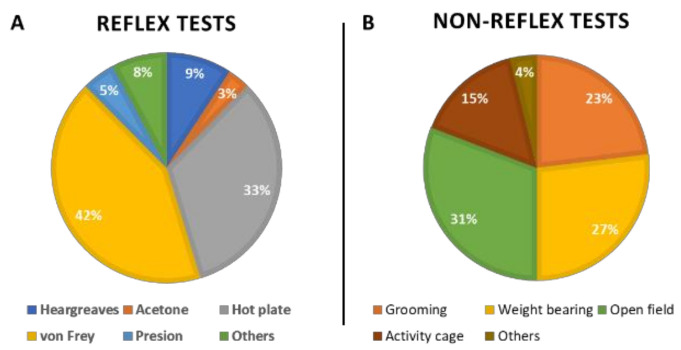
Behavioral tests used to assess endometriosis pain. Most frequent types of reflex (**A**) and non-reflex (**B**) tests used for evaluation of endometriosis-induced pain in rodent models.

Additionally, the main types of behavioral tests used in each category for the evaluation of endometriosis-associated pain were also analyzed (Figure 4) and described in the next section.

#### 2.2.2. Reflex Tests

This group includes the most traditional and widely used tests employed to measure pain in rodents (reflex tests comprise 90% of studies analyzing endometriosis-associated pain). The most common stimuli are mechanical or thermal, and the responses evaluated are latency time, frequency of the response, and pain thresholds. 

In preclinical models for endometriosis, the most used tests to measure hyperalgesia are von Frey for the detection of mechanical thresholds and hot plate for the detection of thermal thresholds (Figure 4A), but other test types were also used for this purpose, as described below. 

##### Mechanical Hyperalgesia

The main tool to measure mechanical hyperalgesia is with a punctate stimulus determined by von Frey filaments [55,75]. In this test, animals are placed in a compartment on a mesh net through which the filaments can be applied to the animals’ test area. The von Frey test has been developed in both manual (monofilaments of calibrated forces) and electronic (one single stimulator includes a force sensor) formats, and the measurement paradigm can be adapted according to the needs of the study. Given that endometriosis-associated pain occurs in the abdominal area, this test should be primarily conducted in that zone. 

Furthermore, if animals also develop generalized pain, it is possible to measure mechanical hyperalgesia on the plantar paw surface [53,56] using either von-Frey filaments or the paw pressure test (also known as Randall-Selitto test), in which rodents receive pressure on the hind paw until showing a struggle response [76]. The main differences between von Frey and Randall-Selitto are (1) the size of the surface area of the stimulation (a small filament in the first vs. a larger, cone shaped surface in the second), and (2) the magnitude of force applied, as the Randall-Selitto test is able to apply much larger magnitudes of force to the stimulated area (technically, von Frey monofilaments could exert a maximum of 52 grams of force (gf) and electronic von Frey devices have a maximum of 100 gf, but Randall-Selitto devices for mice could exert a maximum of 375 gf). This allows for an evaluation of the activity of nociceptors present in tissues deeper than the ones innervating the skin.

Another interesting tool to measure mechanical hyperalgesia in deep tissues is the pressure application measurement (PAM), which is very similar to the Randall-Selitto principle with the advantages that (1) the pressure thresholds are detected and recorded automatically and (2) the pressure applicator can be fixed to the operator’s thumb and be applied in body areas other than the paws. Although this tool has been previously used in rodent models of osteoarthritis [77], it would be interesting to observe the use of this method in the abdominal area of endometriosis-induced animals.

Interestingly, the assessment of vaginal mechanical hyperalgesia has also been studied in preclinical models of endometriosis through a method called vaginal distension [58,59]. It consists of inserting a small balloon intravaginally and applying increasing volumes of pressure until a behavioral escape response is detected from the animals. This method has been established in a rat model of endometriosis performed via the autologous transplantation of uterine tissue. Animals of this model have decreased thresholds of vaginal distension compared to those of sham animals. These findings indicate that the presence of endometriosis lesions may play a role in the development of dyspareunia or pain during sexual intercourse, an important and often neglected symptom in endometriosis patients, and provide a tool for the evaluation of efficacy of potential therapeutic compounds for the treatment of this symptom.

##### Thermal Hyperalgesia

The two most used tests to evaluate thermal hyperalgesia are the Hargreaves and the hot plate test. The first consists of stimulation with a beam of radiant heat to the plantar surface until a withdrawal response is observed [40,61,78]. In the hot plate test, animals are placed on a hot surface with a high temperature (usually between 45 and 55º C) until they show hind paw licking, flicks, or jumping or the cut-off is reached [41,60]. The advantage of the Hargreaves test vs. the hot plate test is that radiant heat application allows for continuous temperature increases on time, allowing for a more sensitive way to detect heat-evoked pain thresholds. One disadvantage for both thermal tests is the desensitization upon repeated heat stimulation, which may lead to confounding results.

Another, less frequently used test is the acetone test, which evaluates cold sensitivity. This test consists of applying a drop of acetone directly onto the ventral surface of the hind paw of the rodent and measuring the time spent licking it [62].

#### 2.2.3. Non-Reflex Tests

Non-reflex tests measure natural behavior in rodents, which can be decreased in pain situations. These kinds of tests were used only minimally in the manuscripts published over the last 25 years; only 19 research articles included non-reflex tests in their studies. This accounted for 30% of the overall number of articles that studied pain in preclinical studies on endometriosis. These tests were mainly analyses of grooming behavior, weight bearing, open field, and in-cage activity (Figure 4B), as detailed below.

##### Pain-like Behavioral Responses

Rather than a single test, this is a group of several tests aimed at evaluating the pain induced by endometriosis by observing different behavioral responses in rodents after model induction, such as abdominal licking, writhing, piloerection, grooming, rearing, and abdominal stretching. These behavioral responses are the most assessed in endometriosis as a non-reflex test using a battery of tests, although not all are usually evaluated in the same study. Responses are evaluated in different ways; for example, licking, writhing and abdominal stretching are measured by the number of times these actions are repeated across a given time [56,63]. Other responses, such as piloerection, are measured according to a previously defined score [64,65]. Another assessment method comprises scoring animals depending on whether they show one, two, or more of these different responses [65]. Moreover, since rodents have inverted circadian rhythms and therefore show highest activity levels during the dark phase, some researchers record behavior in the cages overnight and then analyze these pre-recorded responses without the presence of the researcher in the animal room [79]. 

##### Grooming

This test belongs in the category of the previous paragraph (pain-like responses) but deserves its own section because it is the change in animal behavior evaluated by most authors who measure endometriosis-associated pain by performing a non-reflex test (Figure 3B). Like all non-reflex tests, grooming is a natural activity among rodents, and animals experiencing pain show reduced or inhibited activity [80]. This simple test can help to identify pain located in the visceral area and could be a great tool for the identification of spontaneous pain caused by endometrial lesions in rodents [56]. Spontaneous nociceptive behavior can be evaluated by measuring either the time spent grooming (washing or licking the abdominal area) or by the number of grooming repetitions over a certain period of time, as selected according to the investigator’s discretion [54,63,66]. 

##### Open Field

This test usually is primarily used to measure stress- or anxiety-related behaviors but can also be used as a pain indicator when locomotor activity is decreased [81]. On the one hand, rodents show a natural fear of open areas, but on the other hand, they have an irresistible desire to explore new places. When rodents are under pain states, this exploratory behavior decreases, with a resulting loss of locomotion as they keep closer to the wall. This shows that the time spent in the center and/or near to the wall can be used as an indicator of pain [57,67]: the shorter the time spent in the center and the longer they stay close to the wall, the greater the discomfort experienced by animals. 

##### Locomotor Activity

Exploratory locomotion is a common behavior in rodents. This locomotor activity can dwindle when animals suffer pain, and this decrease in locomotor activity can be used as a pain index [82]. Locomotor activity is measured as total distance travelled for a defined period. Many tests measure this locomotive activity, each with their own particularities (Table 3). Normally these tests are used to assess pain in models with damage to the hind limbs, but they can also be used in models of visceral pain. Table 3 shows the different locomotor activity tests with a brief description.

##### Burrowing and Nesting

Burrowing and nesting are natural activities in highly evolutionarily conserved rodents [85]. These animal instincts can be used to assess pain. The burrowing test consists of a dark tube, open and elevated on one side and filled with gravel or another similar substrate. The animals will burrow in this place, extracting the substrate from the tube as they do in nature, and the extracted substrate is subsequently weighed [69]. 

Nesting is performed by some rodents (mice and rats) for shelter, accommodation, and to maintain temperature. Parameters evaluated include the quality of the nest, the time spent building it, or quantifying the clear areas where material was previously put to build the nest, among those selected by different investigators [53]. 

When animals do not feel well, these behaviors are reduced, and they extract less substrate when burrowing [69] or build worse or even no nests in the nesting test [86]. Since decreased activity in these two tests has been used as an indicator of pain states, these are therefore potentially good tools to identify the presence of visceral pain evoked by endometriosis.

##### Grimace Scale

Pain can induce changes in facial gestures in humans, and these changes can also be identified in rodents [87]. This method is widely used to measure nociception and involves examining facial changes induced by pain stimulation. Although this test has not been reported in our survey of research articles evaluating endometriosis-associated pain, many studies have employed it in visceral pain models [70]. Although expensive equipment is not required, the evaluator must be trained due to the difficulty of identifying these pain gestures. Facial recognition software has recently been developed to make this work faster and more objective [88].

##### Weight Bearing 

The objective of this pain assessment test is to observe changes in rodent posture in response to interference with their physical functioning. Under normal conditions, weight distribution between hind paws in rodents is equal, but pain can induce postural changes that can modify this [89]. This test is usually used to measure weight-bearing changes after damage to one of the hind paws, but some authors have used it in visceral pain models [71,72], in which animals rest more weight on the front paws. As with other visceral pain models, this test could be useful to observe weight distribution differences in animals suffering from endometriosis [73].

Likewise, other models, such as *CatWalk*™ or *Digigait*™, can also be used to evaluate endometriosis-associated pain. In addition to evaluating weight distribution, these systems can measure other parameters, such as swing phases (paw time in the air) and duration of stance (paw time on the floor) [90,91].

##### Skinner Box

This test is not frequently used, but it is an interesting option because it is simple, economical, and gives valuable information. The test comprises an operant chamber with a barrier that the rodent has to climb to reach a reward, such as a sugar pellet. When animals pass through the barrier, pressure increases on the abdomen. If the animals feel abdominal pain, they cross this barrier fewer times compared to that wit healthy animals. This novel test was recently developed to assess endometriosis pain [74]. 

##### Novel-Object Recognition

This test is frequently used to evaluate learning and memory deficits, but changes in nociception sensitivity have also been associated with impaired object recognition in mice [92]. Rodents are curious in nature, so they tend to spend more time around a new object introduced into their cage. When animals feel pain, they employ less time on novel-object recognition [92]. After antinociceptive treatment in other pain models, such as osteoarthritis, animals not only regain the normal threshold for mechanical stimuli, but also experience improved cognitive impairment [93]. Despite the absence of data on preclinical models of endometriosis using novel-object recognition in the literature review, this test has similar usability as other pain models to evaluate endometriosis-associated pain and also the cognitive improvement produced by potential treatments.

## 3. Experimental Section

A literature search was performed using the Medline database to track original research articles published in English about endometriosis, as well as endometriosis and pain, in rodents over the last 25 years (from January 1997 to December 2021). We analyzed the models used to induce endometriosis and the number of these articles studying pain generated by this disease, as well as the type of tests used for this purpose.

### 3.1. Identification of Manuscripts That Studied Endometriosis in Rodents and Model Type Used

The terms used in the Medline searches to determine how many articles have studied endometriosis in rodents was “endometriosis AND (mouse OR mice OR rat OR rodent)”. The result was limited to Title/Abstract, and the reviews were discarded. As some studies in humans contain the words used in the search method in their abstract, a manual review was carried out to discard them. In the same manual review the kind of model used to induce endometriosis was analyzed (see Figure 1).

### 3.2. Identification of Manuscripts That Studied Pain

As in the previous section, to quantify the number of articles studying pain in endometriosis, the terms employed in the Medline database were “endometriosis AND (pain OR nociceptive OR nociception OR analgesia) AND (mouse OR mice OR rat OR rodent)”. The same search parameters as in the previous section were applied, the results were limited to the Title/Abstract, and the reviews were removed. A manual review was performed to eliminate the articles only commenting on but not evaluating the pain in this disease (see Figure 1).

### 3.3. Study of Behavioral Tests Used in Endometriosis Rodent Models

The manual review performed in the previous paragraph to identify pain research was also used to classify whether behavioral tests to measure pain were mediated by reflex or non-reflex stimuli, and each test used was included in each category.

## 4. Conclusions

Endometriosis is a puzzling disease of unclear origin, which commences as a result of genetic, environmental, and immunological factors. About 50% of women with endometriosis suffer pain, and with this review, we only aimed to call attention to the relevance of pain assessments in research in preclinical models. Additionally, we intended to highlight the availability of non-evoked tests, commonly neglected, for evaluation translational research on this disease. Pain is indeed the most universal parameter shared amongst all affected women and the main clinical endpoint of interest. In line with that, pain would be expected to be the most widely tested/explored parameter in research papers where preclinical models are employed to explore the physiopathology of endometriosis. Paradoxically, however, based on our results only a minor fraction (i.e., 6.6%, see Figure 3A) of manuscripts of this kind published in the last 25 years included an evaluation/assessment of endometriosis-associated pain.

Reasons beneath this paradox are uncertain but might rely on the unfamiliarity of researchers in the endometriosis field with pain assessment. Evaluation of pain requires staff trained for such purposes, a research profile that might not be common in endometriosis research groups. Amongst articles assessing pain in rodent models of endometriosis, we detected that most tests used for such purposes were of the reflex type, especially von-Frey and related approaches requiring evoked mechanical/thermal stimuli to achieve a response (Figure 3B). In our view, in the context of endometriosis assessment, there is no rationale for such asymmetry in the infra-use of non-evoked tests. Both reflex and non-reflex tests should be combined for a more powerful and complete evaluation of the pain experience for two main reasons. The first one is that evoked tests can measure nociceptive states in response to “external” stimuli but do not take into consideration the spontaneous pain, which is also a major component of the algesic experience in affected women. In this regard, von-Frey or related evoked tests might be adequate to detect crossenestation and/or altered sensitivity to external tactile stimuli as observed in affected women. Even at some point, evoked tests in animals might cover experiences such dyschezia, dysuria, or dyspareunia, which are also triggered/exacerbated by internal/external stimuli. However, it cannot be neglected that chronic pain experienced by affected women is not related to mechanical stimuli, and therefore, the use of non-evoked tests is an absolute requirement to fulfil and somehow replicate in animals the complex experience of pain in women. This leads to the second reason why pain is not only a simple physiological/physical response but a more complex phenomenon comprising an emotional component, such as distress, anxiety, and depression amongst others. When animals feel pain, they somehow mimic the emotional human experience by stopping in the display of certain innate behaviors [94]. In this context, it is obvious that non-reflex tests are required to assess variations in rodent activity. It must be noted that with certain exceptions [58,71], the use of non-evoked tests to assess pain has been popularized in the last years. This tendency is not restricted to the endometriosis field [43], pointing to the fact that research groups have recognized the importance of studying the emotional component of pain. 

The final goal of these approaches is to enhance the translatability of the animal models to the clinic experience, thus contributing to higher success rates of clinical trials and better treatment options for those who suffer from endometriosis. In our view, enhanced translatability will require three major actions/efforts to be taken in the future as follows: (1) improving the models in terms of replicating physiology (construct-validity); (2) identification of and adequate battery of evoked and non-evoked tests to characterize the pain experience in the model (face-validity); and (3) validation of the model with reference test drugs.

Regarding construct-validity, it is yet undetermined whether a homologous or heterologous model is more likely to better replicate the physiology of human lesions. The advantages and strategies to overcome the disadvantages of both models have been highlighted in this review. Currently, the use of homologous models seems to be favored against heterologous models. The reason for such asymmetry is unclear, but difficulties in accessing human biopsies and the preservation of the immunocompetency might be behind the apparent preference of researchers for the homologous model. As immune cell supplementation techniques improve or endometrial physiology is replicated in rodents, these models will more faithfully mimic what happens in humans.

Regarding face validity, as mentioned above, appropriate modeling will require a combination of both evoked and non-evoked tests. Regarding the latter, despite their utility for pain evaluation, these tests are not widely used yet. At this point, we must call attention to not just the narrow range but the scarce experience with the non-evoked tests employed for endometriosis-induced pain assessment. Indeed, on the list of non-evoked tests potentially available, most of them have only been employed once or twice in the literature. In order to select the most appropriate ones for the purposes of research, we need tests to be popularized and widely assessed so we can compile the experience, debug results, and at the end, choose amongst those being the most sensitive to detect the changes associated with endometriosis induction. As important as establishing an appropriate endometriosis model in rodents, it is necessary to select and establish the appropriate tests to evaluate the study endpoints.

Currently, many groups involved in endometriosis studies are using animal models [45] for the preclinical testing of drugs of interest. In our view, the appropriate modeling of endometriosis must be validated by showing that compound reference drugs used in the clinic [55] are also effective in the animal model. For this, complementing the evoked tests with some non-evoked ones can be useful to discern the analgesic and sedative effect of the drugs, which is not possible with reflex tests.

## Figures and Tables

**Table 2:** Summary of effects of endometriosis or visceral pain on different tests. The number of arrows represents the grade of differences between the group in which pain was induced versus its control group (one arrow means little differences, two arrows medium, and three high differences).

Behavioural Test			Differences vs. Control	Ref.
**Reflex tests**	Mechanical hyperalgesia	Von Frey	↑↑↑	[40,53,55,56,57]
		Randall-Selitto	No data for endometriosis/visceral pain	
		pressure application measurement	No data for endometriosis/visceral pain	
		vaginal distension	↑↑	[58,59]
	Thermal hyperalgesia	Holt plate	↑↑	[41,57,60]
		Hargreaves	↑↑↑	[40,61]
		Acetone	↑↑↑	[62]
**Non-reflex tests**	Pain-like behavioural responses		↑↑↑	[63,64,65]
	Grooming		↑↑	[56,66]
	Open field		↑↑	[57,67]
	Locomotor activity		↑	[53,68]
	Borrowing		↑	[69]
	Nesting		↑↑	[53]
	Grimace scale		↑↑	[66,70]
	Weight bearing		↑↑	[55,71,72,73]
	Skinner box		↑	[74]
	Novel-object recognition		No data for endometriosis/visceral pain	

**Table 3:** Locomotor activity tests.

Test	Brief Description	Ref.
Activity cage	Time and distance spent by animals near walls, in center or in total	[68,83]
Activity wheels	Total distance voluntarily travelled by animals on the wheel	[84]
Automated home cage	Distance travelled and interactions between animals in the same home cage, also during in dark phases when the rodents are more active	[53]

## Data Availability

Not applicable.
